# NPC-EXs Alleviate Endothelial Oxidative Stress and Dysfunction through the miR-210 Downstream Nox2 and VEGFR2 Pathways

**DOI:** 10.1155/2017/9397631

**Published:** 2017-05-28

**Authors:** Hua Liu, Jinju Wang, Yusen Chen, Yanfang Chen, Xiaotang Ma, Ji C. Bihl, Yi Yang

**Affiliations:** ^1^College of Health Science, Wuhan Sports University, Wuhan 430079, China; ^2^Department of Pharmacology & Toxicology, Boonshoft School of Medicine, Wright State University, Dayton, OH 45435, USA; ^3^Guangdong Key Laboratory of Age-Related Cardiac and Cerebral Diseases, Institute of Neurology, Affiliated Hospital of Guangdong Medical University, Zhanjiang 524000, China

## Abstract

We have demonstrated that neural progenitor cells (NPCs) protect endothelial cells (ECs) from oxidative stress. Since exosomes (EXs) can convey the benefit of parent cells through their carried microRNAs (miRs) and miR-210 is ubiquitously expressed with versatile functions, we investigated the role of miR-210 in the effects of NPC-EXs on oxidative stress and dysfunction in ECs. NPCs were transfected with control and miR-210 scramble/inhibitor/mimic to generate NPC-EXs^con^, NPC-EXs^sc^, NPC-EXs^anti-miR-210^, and NPC-EXs^miR-210^. The effects of various NPC-EXs on angiotensin II- (Ang II-) induced reactive oxygen species (ROS) overproduction, apoptosis, and dysfunction, as well as dysregulation of Nox2, ephrin A3, VEGF, and p-VEGFR2/VEGFR2 in ECs were evaluated. Results showed (1) Ang II-induced ROS overproduction, increase in apoptosis, and decrease in tube formation ability, accompanied with Nox2 upregulation and reduction of p-VEGFR2/VEGFR2 in ECs. (2) Compared to NPC-EXs^con^ or NPC-EXs^sc^, NPC-EXs^anti-miR-210^ were less whereas NPC-EXs^miR-210^ were more effective on attenuating these detrimental effects induced by Ang II in ECs. (3) These effects of NPC-EXs^anti-miR-210^ and NPC-EXs^miR-210^ were associated with the changes of miR-210, ephrin A3, VEGF, and p-VEGFR2/VEGFR2 ratio in ECs. Altogether, the protective effects of NPC-EXs on Ang II-induced endothelial injury through miR-210 which controls Nox2/ROS and VEGF/VEGFR2 signals were studied.

## 1. Introduction

Oxidative stress is well-known to play a critical role in a diverse array of cardiovascular disorders including hypertension, diabetic vasculopathy, hypercholesterolemia, and atherosclerosis [1–3]. Indeed, overproduction of reactive oxygen species (ROS) has been shown to induce dysfunction, proinflammatory, and apoptotic death of endothelial cells (ECs) [4–6]. Therefore, an efficient antioxidant defense system to prevent endothelial dysfunction is critical in the protection of vasculature.

Recently, neural crest stem cells have been reported to promote the survival of neurons under normal and oxidative stress conditions in a superoxide dismutase 2 mutant neuron cell line [7]. As one type of stem cells in the brain, neural progenitor cells (NPCs) residing in the subventricular zone contact the blood vessels and directly juxtapose to ECs [8]. The two types of cells could interact with each other through direct physical contact or through paracrine mechanisms with potentially different biological effects. As showed in our previous study [9], NPCs can decrease hypoxia/reoxygenation-induced ROS overproduction on ECs. However, the exact mechanism of NPCs against oxidative stress remains unclear. Exosomes (EXs), small vesicles secreted by most cells, are emerging as mediators for cell-cell communications. They can transfer the carried cargoes such as microRNAs (miRs) and proteins to distant/nearby cells and thereby modulate the recipient cell function [10–12]. Stem cell-derived EXs have been shown to convey the benefits of their parent cells [13–17]. For example, endothelial progenitor cell-derived vesicles can protect ECs against hypoxia/reoxygenation injury [16]. Mesenchymal stem cell-derived EXs can promote functional recovery and neurovascular plasticity after stroke in rats through miR-133 [13] and enhance cell survival in kidney injury [17]. More recently, our group demonstrated that endothelial progenitor cell-derived vesicles from healthy controls have protective effects on the function of endothelial progenitor cells from diabetic patients through their carried miR-126 [14]. In order to explore the mechanism of NPCs protecting ECs from oxidative stress, we investigated whether EXs released from NPCs (NPC-EXs) can protect ECs from oxidative stress and dysfunction in an Ang II-induced injury model.

miR-210 is ubiquitously expressed in a wide range of cells, such as inducible pluripotent stem cells and bone marrow stem cells, and has versatile functions [18]. It is reported that miR-210 is a crucial element of ECs in response to hypoxia which considerably influences the endothelial angiogenic capability [19]. More studies have demonstrated that miR-210 not only influences cell survival by targeting apoptotic genes [20–22] but also displays antioxidant effect by reducing mitochondrial ROS production [23–25]. Recently, Wang et al. reported that EXs derived from inducible pluripotent stem cells can deliver miR-210 to the cardiomyocytes and protect the cardiomyocytes against H_2_O_2_-induced oxidative stress [15]. Nevertheless, there is no study investigating whether miR-210 is involved in the protective effects of NPC-EXs on ECs against oxidative stress.

In this study, we illustrated whether miR-210 participates in the protective effects of NPC-EXs on attenuating Ang II-induced ROS overproduction and dysfunction in ECs.

## 2. Materials and Methods

### 2.1. Cell Culture of NPCs and ECs

Human NPCs were purchased from ATCC® (ATCC-BYS012; Manassas, VA, USA) and cultured according to the manufacturer's protocol. Briefly, NPCs were cultured in complete growth medium which includes DMEM/F12 supplemented with the growth kit for NPC expansion (ATCC ACS-3003; Manassas, VA, USA). Medium was changed every other day. Human brain ECs were purchased from Cell Systems (Kirkland, WA, USA) and cultured with CSC complete medium (Cell Systems) containing 10% serum, 2% human recombinant growth factors, and 0.2% antibiotic solution under standard cell culture conditions (5% CO2, 37°C). Medium was changed twice a week.

### 2.2. Overexpression of miR-210 in NPCs

NPCs were expanded and used for transfection to up- or downregulate of miR-210 [19]. Briefly, the NPCs were cultured to 60–70% confluence and transfected with miR-210 mimic, miR-210 inhibitor, or the scramble control (SC) (40 nM, Exiqon, Woburn, MA) by using lipofectamine 2000 (Invitrogen, Carlsbad, CA) for 24 hrs according to the manufacturer's instruction. NPCs transfected with miR-210 SC, inhibitor, or mimic were denoted as NPCs^sc^, NPCs^anti-miR-210^, or NPCs^miR-210^, respectively. NPCs cultured in complete culture medium served as control (NPCs^con^). The three types of NPCs were used for producing corresponding EXs. All transfections were carried out in triplicate.

### 2.3. Preparation and Collection of EXs Released from NPCs

The protocol for collecting EXs from serum-free culture medium has been reported in our previous study [26]. Briefly, NPCs^con^, NPCs^sc^, NPCs^anti-miR-210^, or NPCs^miR-210^ were cultured in serum-free culture medium to release EXs which were denoted as NPC-EXs^con^, NPC-EXs^sc^, NPC-EXs^anti-miR-210^, and NPC-EXs^miR-210^. After 24 hrs, the medium was collected and centrifuged at 300*g* for 15 mins to remove dead cells. The supernatants were centrifuged at 2000*g* for 30 mins to remove cell debris, followed by centrifugation at 20,000*g* for 70 mins and ultracentrifugation at 170,000*g* for 90 mins to pellet EXs. The pelleted NPC-EXs^con^, NPC-EXs^sc^, NPC-EXs^anti-miR-210^, or NPC-EXs^miR-210^ were resuspended with phosphate-buffered saline (PBS) and aliquoted for nanoparticle tracking analysis (NTA) and coculture experiments. PBS was filtered through 20 nm filter (Whatman, Pittsburgh, PA).

### 2.4. Nanoparticle Tracking Analysis of NPC-EXs

The NanoSight NS300 (Malvern Instruments, Malvern, UK) was used to detect EXs as we previously reported [26]. Briefly, 700 *μ*l diluted suspensions containing NPC-EXs were loaded into the sample chamber and the camera level was maintained at 10 for light scatter mode for sample analysis. Three videos of typically 30-second duration were taken, with a frame rate of 30 frames per second. Data was analyzed by NTA 3.0 software (Malvern Instruments, Malvern, UK) on a frame-by-frame basis. The experiment was repeated four times.

### 2.5. Labeling of NPC-EXs

NPC-EXs were labeled with a red fluorescence dye PKH26 (Sigma Aldrich, St. Louis, MO) as we previously reported [16]. In brief, NPC-EXs were incubated with 2 *μ*M PKH26 dye in PBS for 5 mins at RT. An equal volume of FBS was added to stop staining. Then, NPC-EXs were pelleted by ultracentrifugation and resuspended with culture medium for coculture experiments.

### 2.6. Ang II Injury Model in ECs

ECs were seeded in 12-well plates (5 × 10^4^ cells/well) during the logarithmic growth phase. When 70–80% confluent was reached, the cells were incubated with Ang II (0 or 10^−6^ M; Sigma-Aldrich, St. Louis, MO) for 24 hrs [27]. After coincubation, the medium was replaced with fresh culture medium. ECs were then used for coculture experiments or collected for various analyses.

### 2.7. Coculture of NPC-EXs with ECs

To further elucidate the effects NPC-EXs on ECs, ECs were divided into five coculture groups: vehicle (coculture medium only), NPC-EXs^con^, NPC-EXs^sc^, NPC-EXs^anti-miR-210^, or NPC-EXs^miR-210^. Briefly, the NPC-EXs labeled with PKH 26 were resuspended with CSC medium and added to the culture medium of ECs subjected to Ang II injury. The concentration of NPC-EXs (50 *μ*g/ml) was determined based on our previous study [16]. After 24 hr coculture, the incorporation of NPC-EXs into ECs was observed by fluorescence microscopy (EVOS; Thermo Fisher Scientific). The level of cellular fluorescence intensity was analyzed by ImageJ (NIH) according to the instruction and a previous report [28]. The culture medium of ECs was collected for measuring the concentration of VEGF by ELISA. ECs were used for apoptosis, tube formation, and ROS production assays. The levels of miR-210, ephrin A3, Nox2, and p-VEGFR2/VEGFR2 in ECs were analyzed by quantitative RT-PCR and Western blot, respectively. ECs cultured under normal condition were used as controls. The experiment was repeated for four times.

### 2.8. Apoptosis Assay of ECs

After coculture, ECs were detached for apoptosis assay by using the FITC Annexin V apoptosis detection kit (BD Biosciences, CA) [9]. Briefly, cells were washed with PBS, resuspended with 100 *μ*L 1x annexin-binding buffer, incubated with 5 *μ*L FITC-conjugated annexin V and 5 *μ*L propidium iodide (PI) for 15 mins in the dark, and then analyzed by flow cytometry. The apoptotic cells were defined as annexin V+/PI− cells. The percentage of apoptotic cells was calculated as the following: annexin V+/PI− cells/total cells × 100%. The experiment was repeated four times.

### 2.9. Measurement of ROS

Intracellular ROS production was determined by dihydroethidium (DHE) (Sigma) staining. Briefly, cells were incubated with 2 *μ*M DHE solution at 37°C for 2 hrs. Cells were then washed twice with PBS, and images were taken under a fluorescence microscope (EVOS, Life Sciences). The level of cellular fluorescence intensity was analyzed by ImageJ (NIH, Bethesda, MD) according to a previous report [29]. Data are expressed as fold of fluorescence compared with the control. ECs cultured with CSC medium were used as control.

### 2.10. Quantitative RT-PCR Analysis

After transfection, cells were washed twice with PBS. The miRs from NPCs and their released EXs, as well as the ECs cocultured with various NPC-EXs were extracted using mirVana miRNA isolation kit (Qiagen, Hilden, Germany). For detecting miR-210 level, reverse transcription (RT) reactions were performed by using mirVana qRT-PCR miRNA detection kit and hsa-miR-210 qRT-PCR primer set from Ambion. The RT primer was 5′-GTCGTATCCAGTGCAGGGTCCGAGGTATTCGCACTG GATACGACGACTGT-3′. The forward primer of miR-210 was 5′-CACGCAGTCGTA TCCAGTGCAGG-3′. The reverse primer of miR-210 was 5′-CCAGTGCAGGGTCCG AGGTA-3′. The expression of U6 was used as endogenous control for each sample. The forward primer of U6 was 5′-CTCGCTTCGGCAGCACA-3′, and the reverse primer of U6 was 5′-AACGCTTCACGAATTTGCGT-3′. Relative expression level of each gene was normalized to U6 and calculated using the 2^−ΔΔCT^ method. The experiment was repeated four times.

### 2.11. Enzyme-Linked Immunosorbent Assay

The level of VEGF in the culture medium of ECs cocultured with NPC-EXs^con^, NPC-EXs^sc^, NPC-EXs^anti-miR-210^, or NPC-EXs^miR-210^ were measured by enzyme-linked immunosorbent assay (ELISA) according to the manufacturer's instructions (R&D Systems, Minneapolis, MN, USA). The concentration of VEGF was calculated as pg/ml of culture medium. Each group was triplicated. The experiment was repeated four times.

### 2.12. The Tube Formation Assay for ECs

The tube formation assay was conducted by using in vitro angiogenesis assay kit (Chemicon, Rosemont, IL). First, the ECMatrix solution was thawed and mixed with the ECMatrix diluent. Then, the ECMatrix mixture was placed in a 96-well tissue culture plate at 37°C for 1 hr to allow the matrix solution to solidify. ECs (1 × 10^4^ cells/well) were seeded onto the solidified matrix and incubated at regular cell culture conditions (5% CO2, 37°C). After 24 hr post-seeding, 2 *μ*g/ml calcein (Fisher Scientific, Hampton, NH) was directly added to the culture well and incubated for 20 mins prior to imaging under an inverted fluorescence microscope. Tubes were defined as a tube structure exhibiting a length 4 times of its width [30]. The number of tubes per field was determined. Five random microscopic fields were assessed in each well. The average number from the five fields represents a group.

### 2.13. Western Blot Analysis

After 24 hr treatments, proteins of ECs in different groups were extracted with cell lysis buffer (Thermo Fisher Scientific, Waltham, MA) supplemented with complete mini protease inhibitor tablet (Roche, Basel, Switzerland). Then, the protein lysates were electrophoresed through SDS-PAGE gel and transferred onto PVDF membranes. The membranes were blocked with 5% nonfat milk for 1 hr at room temperature and incubated with primary antibody against ephrin A3 (1 : 500; Abcam, Cambridge, MA), Nox2 (1 : 1000; Abcam, Cambridge, MA), VEGFR2 (1 : 1000; Abcam, Cambridge, MA), p-VEGFR2 (p-Flk1; 1 : 1000; Abcam, Cambridge, MA), or β-actin (1 : 4000; Sigma, St. Louis, MO) at 4°C overnight. On the next day, membranes were washed and incubated with horseradish-peroxidase-conjugated anti-rabbit or anti-mouse IgG (1 : 40,000; Jackson Immuno Research Lab, West Grove, PA) for 1 hr at room temperature. Blots were developed with enhanced chemiluminescence developing solutions, and images were quantified under ImageJ software. The experiment was repeated four times.

### 2.14. Statistical Analysis

All experiments were repeated for four times. Data are expressed as mean ± SEM. Multiple comparisons were analyzed by one- or two-way ANOVA followed by LSD post hoc test. SPSS 17.0 statistical software was used for analyzing the data. For all measurements, a *p* < 0.05 was considered statistically significant.

## 3. Results

### 3.1. Transfection of miR-210 Mimic and Inhibitor Altered the Level of miR-210 in NPCs and NPC-EXs

**Figure 1:**
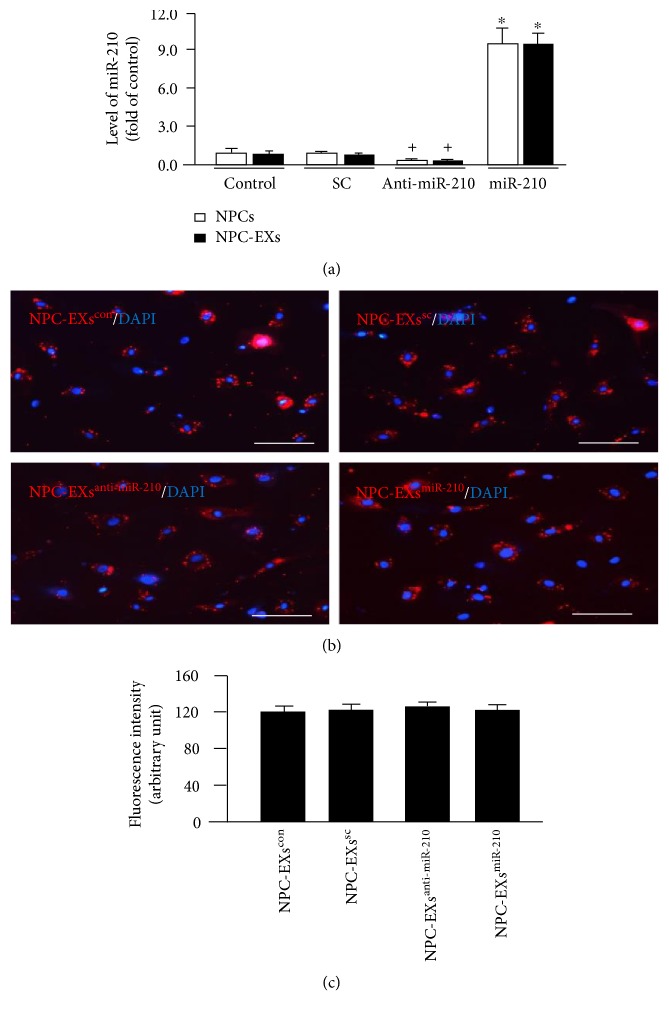
The levels of miR-210 in NPCs and their released NPC-EXs were changed by miR-210 mimic or inhibitor. (a) Summarized data showing the levels of miR-210 in NPCs and NPC-EXs. ^∗^*p* < 0.05 versus control or SC or anti-miR-210; ^+^*p* < 0.05 versus control or SC. SC: scramble control of miR-210. (b) Representative images showing the incorporation of NPC-EXs into the cytoplasm of ECs. (c) Summarized data showing the fluorescence intensity in ECs. Data are expressed as mean ± SEM. *N* = 4/group.

Upon the results obtained from NTA analysis (Table 1), modulation of miR-210 did not change the size and the concentration of EXs released from NPCs (versus NPC-EXs^con^ or NPC-EXs^sc^, *p* > 0.05). As shown in Figure 1(a), transfection of the miR-210 mimic significantly increased miR-210 level in NPCs. As expected, miR-210 level was higher in NPC-EXs^miR-210^ than that in NPC-EXs^con^ and NPC-EXs^sc^. On the contrary, miR-210 inhibitor significantly decreased the level of miR-210 in NPCs and their released EXs.

### 3.2. NPC-EXs^miR-210^ Significantly Upregulated the Level of miR-210 and Downregulated Ephrin A3 Level in ECs

As shown in Figures 1(b) and 1(c), PKH26-labeled NPC-EXs were observed in the cytoplasm of ECs after 24 hr of coculture, indicating EXs were uptaken by ECs. There was no significant difference of the fluorescence intensity among different groups.

**Figure 2:**
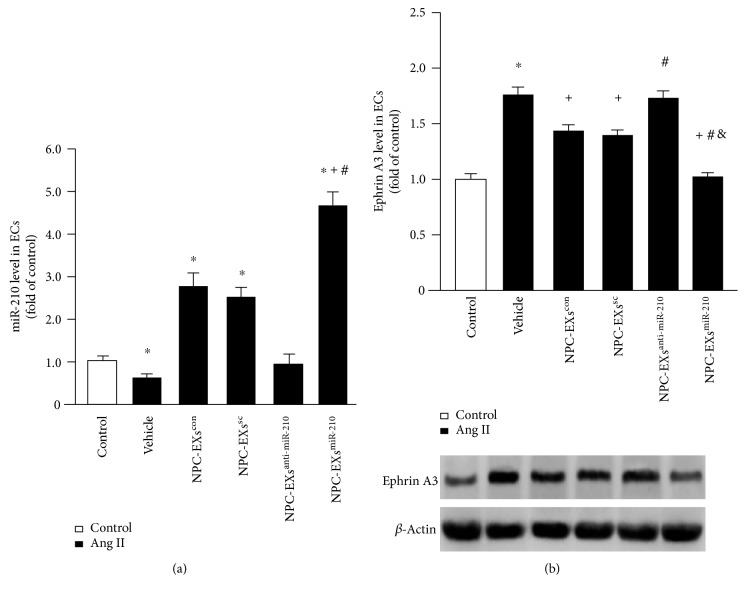
Up- or downregulation of miR-210 affected the effects of NPC-EXs on the levels of miR-210 and its target gene ephrin A3. (a) The level of miR-210 in ECs treated with various NPC-EXs. (b) Summarized data and representative bands showing the protein level of ephrin A3 in ECs treated with various NPC-EXs. ^∗^*p* < 0.05 versus control; ^+^*p* < 0.05 versus vehicle; ^#^*p* < 0.05 versus NPC-EXs^con^ or NPC-EXs^sc^; ^&^*p* < 0.05 versus NPC-EXs^anti-miR-210^. EXs released from NPCs^con^, NPCs^sc^, NPC-EXs^anti-miR-210^, or NPCs^miR-210^ were denoted as NPC-EXs^con^, NPC-EXs^sc^, NPC-EXs^anti-miR-210^, or NPC-EXs^miR-210^. Data are expressed as mean ± SEM. *N* = 4/group.

In order to evaluate whether coculture with NPC-EXs can alter the level of miR-210 in ECs, we conducted quantitative RT-PCR to determine the level of miR-210 in ECs after different treatments. As shown in Figure 2(a), Ang II downregulated the level of miR-210 in ECs (versus control, *p* < 0.05). In the treatment groups, coculture with NPC-EXs^con^ or NPC-EXs^sc^ alone significantly upregulated miR-210 level in ECs (versus vehicle, *p* < 0.05). NPC-EXs^anti-miR-210^ did not significantly alter the level of miR-210 in Ang II-injured ECs (versus vehicle, *p* > 0.05), whereas, NPC-EXs^miR-210^ remarkably raised the level of miR-210 in ECs (versus NPC-EXs^con^ or NPC-EXs^sc^ or NPC-EXs^anti-miR-210^, *p* < 0.05). These data suggest that NPC-EXs can deliver miR-210 into ECs in vitro. We also determined the protein level of ephrin A3, a target gene of miR-210, in ECs after coculture. Our data (Figure 2(b)) showed that the ephrin A3 level was upregulated in Ang II-injured ECs (versus control, *p* < 0.05), which was downregulated by NPC-EXs^con^ or NPC-EXs^sc^ coculture (versus vehicle, *p* < 0.05). NPC-EXs^anti-miR-210^ did not significantly affect, while NPC-EXs^miR-210^ upregulated the level of ephrin A3 in Ang II-injured ECs. These findings reflect that NPC-EXs and NPC-EXs^miR-210^ could transfer miR-210 to ECs and modulate the expression of its target gene, ephrin A3, in ECs.

### 3.3. NPC-EXs^anti-miR-210^ Were Less, Whereas NPC-EXs^miR-210^ Were More Effective on Reducing Ang II-Induced Apoptosis, ROS Overproduction, and Nox2 Upregulation on ECs

**Figure 3:**
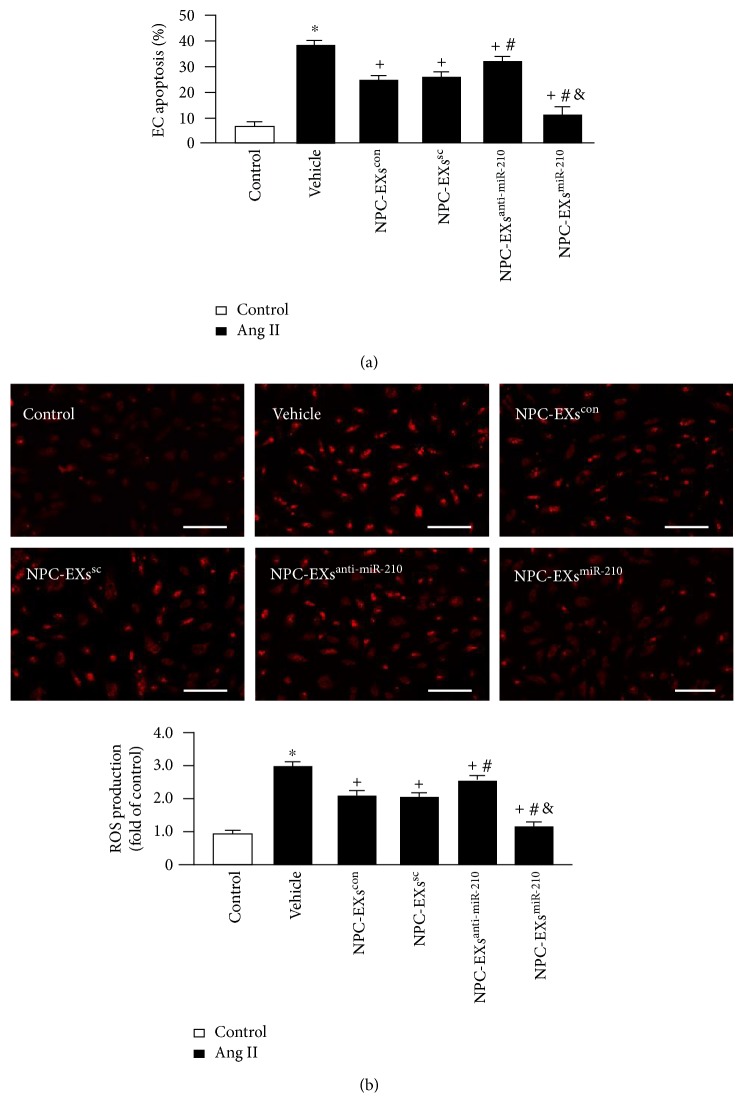
Up- or downregulation of miR-210 modulated the effects of NPC-EXs on reducing Ang II-induced apoptosis and ROS overproduction in ECs. (a) The apoptotic rate of ECs treated with different NPC-EXs. (b) Up: representative images showing DHE staining of ECs treated with different NPC-EXs; bar: 100 *μ*m; bottom: summarized data; ^∗^*p* < 0.05 versus con; ^+^*p* < 0.05 versus vehicle; ^#^*p* < 0.05 versus NPC-EXs^con^ or NPC-EXs^sc^; ^&^*p* < 0.05 versus NPC-EXs^anti-miR-210^. EXs released from NPCs^con^, NPCs^sc^, NPC-EXs^anti-miR-210^, or NPCs^miR-210^ were denoted as NPC-EXs^con^, NPC-EXs^sc^, NPC-EXs^anti-miR-210^, or NPC-EXs^miR-210^. Data are expressed as mean ± SEM. *N* = 4/group.

To verify the model of Ang II-induced EC injury, we conducted flow cytometry and DHE staining to evaluate the apoptotic rate and ROS production, respectively. Our results (Figure 3) showed that Ang II significantly increased the percentage of ECs in the early apoptosis phase and the level of ROS production (versus control, *p* < 0.05).

To determine the effects of NPC-EXs on ECs against Ang II-induced oxidative stress injury, we cocultured Ang II-injured ECs with various NPC-EXs. Our data showed that coculture with NPC-EXs^con^ or NPC-EXs^sc^ alone can reduce Ang II-induced apoptosis and ROS overproduction (versus vehicle, *p* < 0.05). NPC-EXs^anti-miR-210^ can decrease these detrimental effects of Ang II, but was less than NPC-EXs^con^ and NPC-EXs^sc^ did (versus NPC-EXs^con^ or NPC-EXs^sc^, *p* < 0.05). NPC-EXs^miR-210^ had the most effects on decreasing Ang II-induced apoptosis and ROS production of ECs among the four types of NPC-EXs.

**Figure 4:**
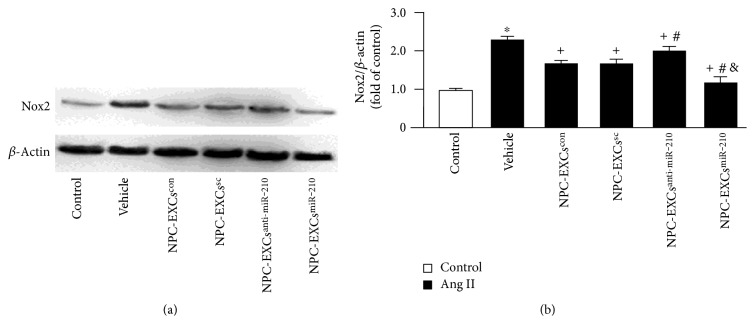
Manipulation of miR-210 with inhibitor or mimic altered the effect of NPC-EXs on reducing Ang II-induced Nox2 expression. (a) Representative Western blot bands. (b) Summarized data showing the expression of Nox2 in ECs treated with different NPC-EXs; ^∗^*p* < 0.05 versus con; ^+^*p* < 0.05 versus vehicle; ^#^*p* < 0.05 versus NPC-EXs^con^ or NPC-EXs^sc^; ^&^*p* < 0.05 versus NPC-EXs^anti-miR-210^. EXs released from NPCs^con^, NPCs^sc^, NPC-EXs^anti-miR-210^, or NPCs^miR-210^ were denoted as NPC-EXs^con^, NPC-EXs^sc^, NPC-EXs^anti-miR-210^, or NPC-EXs^miR-210^. Data are expressed as mean ± SEM. *N* = 4/group.

In addition, we analyzed the expression of Nox2 in ECs after cocultured with different NPC-EXs. Our Western blot results (Figure 4) showed that Ang II upregulated Nox2 expression in ECs (versus control, *p* < 0.05). Coculture with NPC-EXs^con^ or NPC-EXs^sc^ alone significantly downregulated Nox2 expression (versus vehicle, *p* < 0.05). NPC-EXs^anti-miR-210^ can downregulate Nox2 expression, but had much less effect than NPC-EXs^con^ and NPC-EXs^sc^ had (versus NPC-EXs^con^ or NPC-EXs^sc^, *p* < 0.05). Similarly, NPC-EXs^miR-210^ had the most effect on decreasing Ang II-induced Nox2 expression in ECs.

Collectively, this data suggests that miR-210 plays a role in the protective effects of NPC-EXs on ECs against Ang II-induced oxidative stress, and exogenous miR-210 can boost the antioxidant and antiapoptosis effects elicited by NPC-EXs.

### 3.4. NPC-EXs^anti-miR-210^ Were Less, Whereas NPC-EXs^miR-210^ Were More Effective on Increasing VEGF Secretion and Improving the Tube Formation Ability of ECs

**Figure 5:**
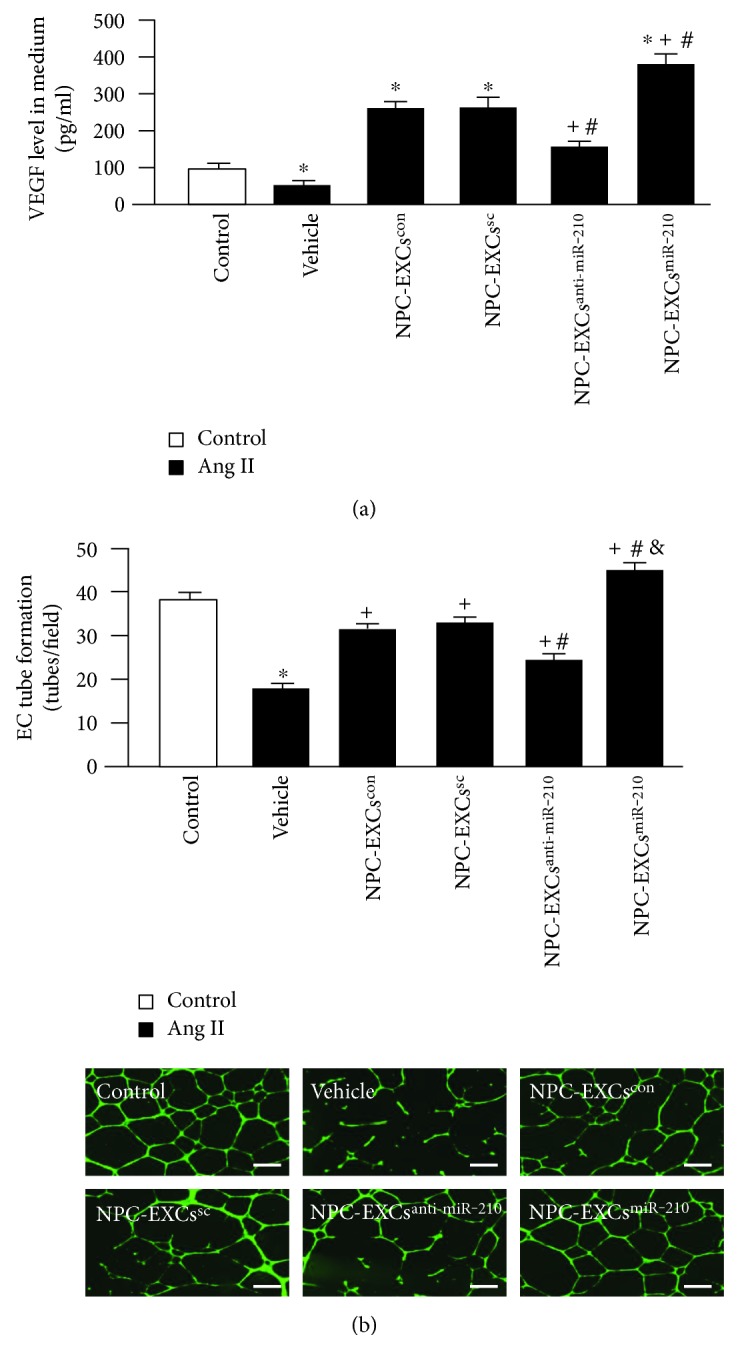
Modulation of miR-210 altered the effect of NPC-EXs on improving the VEGF secretion and tube formation ability of ECs. (a) VEGF level in the culture medium of ECs treated with various NPC-EXs. (b) Summarized data of tube formation and representative images showing the tube formation of ECs treated by various NPC-EXs; bar: 500 *μ*m. ^∗^*p* < 0.05 versus control; ^+^*p* < 0.05 versus vehicle; ^#^*p* < 0.05 versus NPC-EXs^con^ or NPC-EXs^sc^; ^&^*p* < 0.05, versus NPC-EXs^anti-miR-210^. Data are expressed as mean ± SEM. *N* = 4/group.

In order to assess whether NPC-EXs can improve the endothelial angiogenic function compromised by Ang II, we analyzed VEGF level in the culture medium and the tube formation ability of ECs. Our data showed that the level of VEGF in the culture medium was decreased in ECs injured by Ang II (versus control, *p* < 0.05). In the treatment groups, coculture with NPC-EXs^con^ and NPC-EXs^sc^ increased the VEGF level (versus vehicle, *p* < 0.05), whereas NPC-EXs^anti-miR-210^ had less effect than NPC-EXs^con^ and NPC-EXs^sc^ had (versus NPC-EXs^con^ or NPC-EXs^sc^, *p* < 0.05). Similarly, NPC-EXs^miR-210^ had the most effect on upregulating VEGF secretion of ECs among the four types of NPC-EXs (Figure 5(a)).

Similarly, the tube formation ability of ECs was compromised by Ang II (versus control, *p* < 0.05). Coculture with NPC-EXs^con^ or NPC-EXs^sc^ alone significantly improved the tube formation ability of ECs subjected to Ang II (versus vehicle, *p* < 0.05). NPC-EXs^anti-miR-210^ displayed less effect than NPC-EXs^con^ or NPC-EXs^sc^ did. Likely, NPC-EXs^miR-210^ had the most effect on promoting tube formation (Figure 5(b)). Taken together, these results indicate that miR-210 is involved in the effect elicited by NPC-EXs on improving the angiogenic function of Ang II-injured ECs.

### 3.5. NPC-EXs^anti-miR-210^ Were Less, Whereas NPC-EXs^miR-210^ Were More Effective on Upregulating the Expression of p-VEGFR2/VEGFR2 in ECs

**Figure 6:**
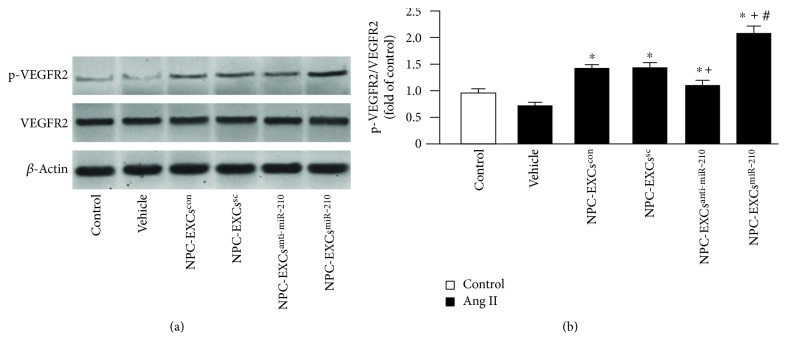
Up- and downregulation of miR-210 altered the effect of NPC-EXs on the expression of p-VEGFR2/VEGFR2 in ECs. (a) Representative western blot bands. (b) Summarized data. ^∗^*p* < 0.05 versus vehicle; ^+^*p* < 0.05 versus NPC-EXs^con^ or NPC-EXs^sc^; ^#^*p* < 0.05 versus NPC-EXs^anti-miR-210^. Data are expressed as mean ± SEM. *N* = 4/group.

As shown in Figure 6, the ratio of p-VEGFR2/VEGFR2 in ECs was decreased by Ang II (versus control, *p* < 0.05). In the treatment groups, coculture with NPC-EXs^con^ or NPC-EXs^sc^ increased the phosphorylation of VEGFR2 (versus control or vehicle, *p* < 0.05). NPC-EXs^anti-miR-210^ also raised the expression ratio of p-VEGFR2/VEGFR2, but the effect was less than NPC-EXs^con^ or NPC-EXs^sc^ had (versus NPC-EXs^con^ or NPC-EXs^sc^, *p* < 0.05). Similarly, NPC-EXs^miR-210^ had the strongest effect on upregulating the ratio of p-VEGFR2/VEGFR2 in ECs subjected to Ang II injury (versus NPC-EXs^con^, NPC-EXs^sc^, or NPC-EXs^anti-miR-210^, *p* < 0.05).

## 4. Discussion

In the present study, we have demonstrated that NPC-EXs have protective effects against Ang II-induced oxidative stress, cell death, and angiogenic dysfunction in ECs, which are at least partly through Nox2/ROS and VEGF/VEGFR2 signaling pathways modulated by miR-210.

Oxidative stress is the major cause of endothelial dysfunction which is implicated in various vascular diseases [31]. Ang II has been well documented to trigger ROS overproduction and decrease the production/bioavailability of nitric oxide of ECs, which consequently contribute to endothelial dysfunction [32, 33]. In this study, we used Ang II to construct an EC oxidative stress model. As agreed with previous reports [27, 34], Ang II resulted in increasing of apoptosis, overproduction of ROS, and impairment of angiogenic function (tube formation ability and VEGF secretion), along with the upregulation of Nox2 level and downregulation of the ratio of p-VEGFR2/VEGFR2 in ECs.

We previously demonstrated that NPCs could decrease apoptosis and ROS overproduction on hypoxia/reoxygenation-injured ECs [9]. Our study is supported by a recent report showing that neural crest-derived stem cells promote the survival of neurons under normal and oxidative stress conditions in a mutant neuron cell line [7]. However, the underlying mechanism requires further investigation. Currently, EXs are emerging as cell-cell communicators. Mounting evidence shows that stem cell-derived EXs harbor the benefits of their parent cells and can alter the function of recipient cells [13, 16, 17]. For instance, mesenchymal stem cell-derived extracellular vesicles can enhance cell survival in kidney injury [17] and promote functional recovery and neurovascular plasticity after stroke in rats [13]. We have demonstrated that endothelial progenitor cell-derived vesicles can protect ECs against hypoxia/reoxygenation [16]. In this study, we investigated whether NPC-EXs could rescue injured ECs following direct oxidative stress by using the Ang II injury model. In order to test our hypothesis, we cocultured NPC-EXs^con^ with Ang II-injured ECs and found that NPC-EXs^con^ could be uptaken by ECs and can attenuate Ang II-induced apoptosis, ROS overproduction, and Nox2 upregulation. In addition, the tube formation capacity and VEGF secretion capacities compromised by Ang II were also rescued by NPC-EXs^con^. All of these findings suggest that NPC-EXs can protect ECs against Ang II, associated with the suppression of Nox2 expression and subsequently inhibiting ROS overproduction.

Interestingly, we also found that the level of miR-210 was increased in ECs after coincubation with NPC-EXs. This raised the hypothesis that the protective effects of NPC-EXs on Ang II-induced ECs might be related with miR-210. Previous studies have demonstrated that miR-210 is ubiquitously expressed in a wide range of cells and displays versatile functions such as antioxidative stress [15, 25, 35, 36] and apoptosis defense [20–22]. Blockade of miR-210 in myoblasts greatly increases myoblast cell sensitivity to oxidative stress and mitochondrial dysfunction [25]. Overexpression of miR-210 enhances the survival of mesenchymal stem cells in an oxidative stress environment [35] and reduces ROS overproduction as well as cardiomyocyte death in response to hypoxia-reoxygenation [36]. Recently, Wang et al. reported that EXs derived from inducible pluripotent stem cells can deliver miR-210 to cardiomyocytes and protect them against H_2_O_2_-induced oxidative stress [15]. In order to investigate the potential mechanisms, we tested our hypothesis that miR-210 modulates the beneficial effects exhibited by NPC-EXs on Ang II-injured ECs. First, we up- or downregulated miR-210 in NPCs and found that the miR-210 level was parallelly raised or reduced in their corresponding EXs, NPC-EXs^miR-210^, and NPC-EXs^anti-miR-210^. This suggests that NPCs carried by miR-210 was packaged into NPC-EXs. Next, we cocultured NPC-EXs^miR-210^, NPC-EXs^anti-miR-210^, and their controls (NPC-EXs^sc^) with Ang II-injured ECs. Our data showed that both NPC-EXs^sc^ and NPC-EXs^miR-210^ increased the miR-210 level in ECs. Meanwhile, we found that the protein level of ephrin A3, a target gene of miR-210 [19], was significantly downregulated by NPC-EXs^miR-210^ in Ang II-injured ECs. These results reflect that NPC-EXs can deliver miR-210 into ECs. Besides, we found that NPC-EXs^miR-210^ had the strongest effects on decreasing Ang II-induced apoptosis and ROS production, indicating miR-210 can boost the protection effects of NPC-EXs on ECs. As one of the most prominent sources of vascular ROS [37], Nox2 level was also evaluated. We found the Nox2 level in ECs was remarkably decreased by NPC-EXs^miR-210^, suggesting that the antioxidant effect of NPC-EXs might be associated with the Nox2/ROS signal. In contrast, NPC-EXs^anti-miR-210^ partially reduced the protective effects of NPC-EXs, which further confirmed the important role of miR-210 in NPC-EXs for protecting ECs from Ang II-induced injury. This data also suggests that other cargoes such as proteins and cytokines carried by NPC-EXs might also participate in the protection event.

As we know, VEGF is a proangiogenic factor [38] which could be modulated by ephrin A3 [39]. In the present study, our data indicated that NPC-EXs^miR-210^ could decrease ephrin A3 level whereas increase VEGF level in the culture medium of ECs, suggesting that ephrin A3 is connecting miR-210 and VEGF in ECs. The result showing the increase of VEGF level is coherent with that NPC-EXs^miR-210^ improved tube formation ability of ECs compromised by Ang II. VEGFR2 is responsible for most downstream angiogenic effects of VEGF [40]. Binding of VEGF to VEGFR2 can activate downstream survival and migration pathways involving PI3-kinase/Akt and focal adhesion kinase, respectively [41]. In order to determine whether the increased level of VEGF can activate VEGFR2 signal, we used Western blots to evaluate the changes in the signaling proteins VEGFR2 and its phosphorylation. We found the total expression of VEGFR2 in ECs was not changed after NPC-EX treatment, while the expression of p-VEGFR2 was remarkably increased by NPC-EXs^miR-210^, indicating the activation of the VEGF/VEGFR2 signal. Meanwhile, our data indicated that NPC-EXs^anti-miR-210^ also upregulated the phosphorylating VEGFR2, though at a less extent as compared to that of NPC-EXs^con^ or NPC-EXs^sc^ had. This effect could be resulted from other cargoes such as proteins and cytokines carried by NPC-EXs^anti-miR-210^. Collectively, these data reflect that miR-210 can modulate the effect of NPC-EXs on ECs subjected to Ang II injury by activating the VEGF/VEGFR2 signal.

## 5. Conclusion

Our results demonstrate that miR-210 can modulate the effects of NPC-EXs on protecting ECs from Ang II-induced oxidative stress, majorly through the Nox2/ROS and VEGF/VEGFR2 signals.

## Figures and Tables

**Table 1:** NTA analysis of the size and concentration of various NPC-EXs.

EX type	Size range (nm)	Concentration (×10^6^ particles/ml)
NPC-EXs^con^	102 ± 30	5.67 ± 0.07
NPC-EXs^sc^	104 ± 43	5.58 ± 0.11
NPC-EXs^anti-miR-210^	100 ± 51	5.69 ± 0.18
NPC-EXs^miR-210^	105 ± 62	5.74 ± 0.08

NPC-EXs^con^, NPC-EXs^sc^, NPC-EXs^anti-miR-210^, and NPC-EXs^miR-210^ represent EXs released from NPCs cultured in culture medium (control) and transfected with scramble control, miR-210 inhibitor, or miR-210 mimic. Data are expressed as mean ± SEM. *N* = 4/group.
