# EXPRESSION OF CONCERN: NPC‐EXs Alleviate Endothelial Oxidative Stress and Dysfunction through the miR‐210 Downstream Nox2 and VEGFR2 Pathways

**DOI:** 10.1155/omcl/9891762

**Published:** 2026-07-10

EXPRESSION OF CONCERN: H. Liu, J. Wang, Y. Chen, et al., “NPC‐EXs Alleviate Endothelial Oxidative Stress and Dysfunction through the miR‐210 Downstream Nox2 and VEGFR2 Pathways,” *Oxidative Medicine and Cellular Longevity*, 2017, 9397631, https://doi.org/10.1155/2017/9397631.

This Expression of Concern is for the above article, published online on 28 May 2017 in Wiley Online Library (wileyonlinelibrary.com), and has been issued by agreement between the Journal Editor‐in‐Chief; Jeannette Vasquez‐Vivar; and John Wiley & Sons Ltd. The Expression of Concern has been agreed due to concerns reported on PubPeer regarding Figure 5b [1]. Specifically, a region of the control image in Figure 5b appears to overlap with a region of the GSC‐EXsmiR‐21 image in Figure 5a of a previous publication with three overlapping authors from this article [2], published on 29 March 2017.

The authors maintain that the images in the present article are all correct, and state that the image in Figure 5a of [2] is incorrect due to an error introduced by mislabelling of the image in a shared data folder.

Because of potential confusion raised by the PubPeer comment, the journal has decided to issue an Expression of Concern to inform and alert readers. The authors have been informed of the Expression of Concern.
